# The effect of national antenatal care guidelines and provider training on obstetric danger sign counselling: a propensity score matching analysis of the 2014 Ethiopia service provision assessment plus survey

**DOI:** 10.1186/s12978-022-01442-6

**Published:** 2022-06-06

**Authors:** Tebikew Yeneabat, Andrew Hayen, Theodros Getachew, Angela Dawson

**Affiliations:** 1 Department of Midwifery, College of Health Sciences, Debre Markos University, Debre Markos, Ethiopia; 2 School of Public Health, Faculty of Health, University of Technology Sydney, Sydney, Australia; 3 Health System and Reproductive Health Research Directorate, Ethiopian Public Health Institute, Addis Ababa, Ethiopia

**Keywords:** Antenatal care, Counselling, Obstetric danger signs

## Abstract

**Background:**

Most pregnant women in low and lower-middle-income countries do not receive all components of antenatal care (ANC), including counselling on obstetric danger signs. Facility-level ANC guidelines and provider in-service training are major factors influencing ANC counselling. In Ethiopia, little is known about the extent to which guidelines and provider in-service training can increase the quality of ANC counselling.

**Methods:**

We examined the effect of national ANC guidelines and ANC provider in-service training on obstetric danger sign counselling for pregnant women receiving ANC using the 2014 Ethiopian service provision assessment plus (ESPA +) survey data. We created two analysis samples by applying a propensity score matching method. The first sample consisted of women who received ANC at health facilities with guidelines matched with those who received ANC at health facilities without guidelines. The second sample consisted of women who received ANC from the providers who had undertaken in-service training in the last 24 months matched with women who received ANC from untrained providers. The outcome variable was the number of obstetric danger signs described during ANC counselling, ranging from zero to eight. The covariates included women’s socio-demographic characteristics, obstetric history, health facility characteristics, and ANC provider characteristics.

**Results:**

We found that counselling women about obstetric danger signs during their ANC session varied according to the availability of ANC guidelines (61% to 70%) and provider training (62% to 68%). After matching the study participants by the measured covariates, the availability of ANC guidelines at the facility level significantly increased the average number of obstetric danger signs women received during counselling by 24% (95% CI: 12–35%). Similarly, providing refresher training for ANC providers increased the average number of obstetric danger signs described during counselling by 37% (95% CI: 26–48%).

**Conclusion:**

The findings suggest that the quality of ANC counselling in Ethiopia needs strengthening by ensuring that ANC guidelines are available at every health facility and that the providers receive regular ANC related in-service training.

**Supplementary Information:**

The online version contains supplementary material available at 10.1186/s12978-022-01442-6.

## Plain language summary

Maternal death from preventable pregnancy-related complications remains a global health challenge. In 2017, there were 295,000 maternal deaths worldwide, and about two-thirds of these deaths were from Sub-Saharan Africa. Ethiopia is a Sub-Saharan African country with 401 maternal deaths per 100,000 live births in 2017, and this rate is higher than the target indicated in sustainable development goals. Most maternal deaths are due to obstetric complications and could have been averted through early detection and treatment. Providing antenatal care counselling about obstetric danger signs enhances women’s awareness of obstetric complications and encourages women to seek treatment from a skilled care provider. However, most women from low-income settings, including Ethiopia, do not receive counselling about obstetric danger signs. Facility-level antenatal care guidelines and provider in-service training improve antenatal care counselling. In Ethiopia, little is known to what extent antenatal care guidelines and provider training increase counselling on obstetric danger signs. The present study used the 2014 Ethiopian service provision assessment data and estimated the effect of antenatal care guidelines and provider training on counselling about obstetric danger signs. The analysis involved a propensity score matching method and included 1725 pregnant women. The study found that antenatal care guidelines at health facilities and antenatal care provider in-service training significantly increase counselling on obstetric danger signs by 24% and 37%, respectively. The finding suggests improving the quality of antenatal care counselling in Ethiopia needs antenatal care guidelines at each antenatal care clinic and refresher training for the providers.

**Supplementary Information:**

The online version contains supplementary material available at 10.1186/s12978-022-01442-6.

## Background

Maternal death, the death of a woman while pregnant, during childbirth, or within 42 completed days of termination of pregnancy [1], remains a global health challenge [2, 3]. It has a negative effect on infant and child survival, the well-being of the family and society, and the country's socio-economic development by increasing health costs and reducing productivity [4–6]. As a result, improving maternal health and reducing maternal mortality is key to achieving sustainable development goals (SDGs). Sustainable development goal target 3 includes reducing the maternal mortality ratio to less than 70 per 100,000 live birth by 2030 [7–9].

Between 2000 and 2017, there was a 38% decline in the global maternal mortality ratio (MMR). In 2017 there were 295,000 maternal deaths, with an estimated 211 maternal deaths per 100,000 live births. This rate is higher than the SDG target. About 94% of these maternal deaths were from low-income countries. Sub-Saharan Africa (SSA) alone had 196,000 maternal deaths, accounting for nearly two-thirds of the global maternal deaths [2, 3, 8]. Ethiopia is an SSA country where maternal deaths have remained very high, particularly between 2000 and 2011. There were 871, 673 and 676 maternal deaths per 100,000 live births in 2000, 2005 and 2011, respectively [10–12]. As a result, the Ethiopian Ministry of Health has prioritised maternal health. The Ministry of Health launched the health sector development program (HSTP) in 2015 that outlined strategies to provide quality healthcare [13]. The main focus of the HSTP is to ensure universal health coverage by strengthening primary healthcare [13], including training and deploying community health extension workers [14]. As a result, the country has made remarkable progress in reducing MMR from 676 in 2011 to 412 in 2016 [15]. A recent report indicated that MMR in Ethiopia was 401 in 2017 [3], which has declined by slightly more than half compared to 871 in 2000 [10]. However, the current MMR in Ethiopia is considerably high as it surpasses the SDGs target of no more than 140 maternal deaths per 100,000 live births, and interventions need to be scaled up to avert preventable maternal deaths [7, 8].

Most maternal deaths are preventable through quality healthcare services, such as antenatal care (ANC) [3]. For example, up to half of all maternal deaths can be prevented through ANC that provides comprehensive counselling on obstetric danger signs and facilitates the early detection and timely management of pregnancy-related complications and other pre-existing health problems [3, 16–19]. Improving pregnancy outcomes requires a pregnant woman to commence her first ANC contact at or before 16 weeks of gestation and to receive the recommended components of care, including ANC counselling on obstetric danger signs that enhance risk identification [20–22]. Antenatal care counselling on obstetric danger signs enhances a woman’s awareness of obstetric complications and encourages treatment-seeking from a skilled care provider during pregnancy, childbirth and postpartum [23].

Studies showed that ANC counselling, an interaction between the ANC provider and a woman and her family involving the exchange of information providing support [24], regarding pregnancy-related topics, such as obstetric danger signs, improves maternal health literacy about obstetric complications contributing to a women’s decision to seek timely treatment ([25] Under review), skilled birth attendance [26] and postpartum family planning [27, 28]. An example of an interactive ANC counselling approach is group ANC (GANC), a method of pregnancy care that provides eight to 12 women the opportunity to share their pregnancy and childbirth life experiences and learn from peers and the care provider. GANC has been found to enhance a woman’s awareness of obstetric danger signs [29–31]. However, most women do not receive ANC counselling on obstetric danger signs [17, 32–35]. ANC counselling in some low and lower-middle-income countries is as low as 13% ([25] Under review). In Ethiopia, only 45% of women who received ANC were counselled on obstetric danger signs [15].

Factors relating to a woman’s demand for health care and the supply of health services can influence the quality of ANC counselling. Examples of demand-related factors include a woman’s educational level [36] and the number of ANC contacts she has [16, 35, 37]. Supply related factors are primarily human and material resources [20, 23, 38–40]. However, the lion’s share of factors influencing quality ANC counselling are facility-level ANC guidelines and ANC provider uptake of in-service training [29, 41–43].

The importance of ANC guidelines and provider in-service training in improving ANC counselling on obstetric danger has been reported in studies from Benin [42], Guatemala [43] and Tanzania [41]. In Ethiopia, however, little is known to what extent guidelines and in-service training increase ANC counselling on obstetric danger signs. Therefore, the present study aimed to answer the research question “Does the availability of national ANC guidelines at the facility level and ANC in-service training in the last 24 months affect antenatal counselling on obstetric danger signs?”. The study estimates the extent to which the availability of facility-level national ANC service guidelines and trained ANC providers can increase ANC counselling on obstetric danger signs in Ethiopia.

## Methods

### Study design and data source

This is a cross-sectional study based on the 2014 Ethiopian service provision assessment plus (ESPA +) survey data [44]. The 2014 ESPA + was the first nationally representative facility-based survey on the performance of health facilities in Ethiopia. The 2014 ESPA + survey utilised four data collection instruments: facility inventory questionnaires, provider interview questionnaires, client exit interviews, and observation checklists of client-provider interactions. Facility inventory questionnaires were used to collect data on the availability of services, medicines, infrastructures, and supplies. The provider interview questionnaires were used to collect data on the service environment and the healthcare provider’s practices and perspectives (experiences and qualifications). Observation checklists were used to assess the extent to which the provider applied the accepted standards of care. The client exit interview questionnaires were used to collect data on the quality of client-provider interaction [44].

### Sample size and selection process

**Fig. 1:**
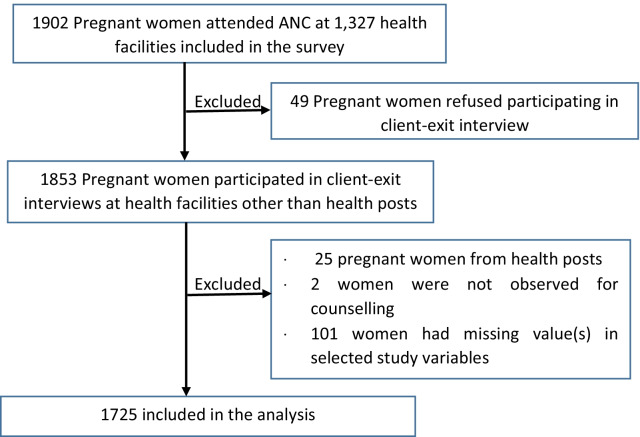
Sample selection process (unweighted sample)

The 2014 ESPA + survey data involved 1902 women receiving ANC at 1327 health facilities [44]. We excluded 177 women from the analysis for the following reasons (see Fig. 1). Forty-nine women refused to participate in the client-exit interview. Twenty-five women attended health posts, the lowest health facilities at the primary healthcare level staffed with health extension workers [14] who are not skilled attendants, as defined by the World Health Organization (WHO) [45]. Two women were not observed receiving ANC counselling on obstetric danger signs. One hundred one women had missing values in one or more study variables. Finally, we included 1725 women in this analysis.

### Study variables

**Fig. 2:**
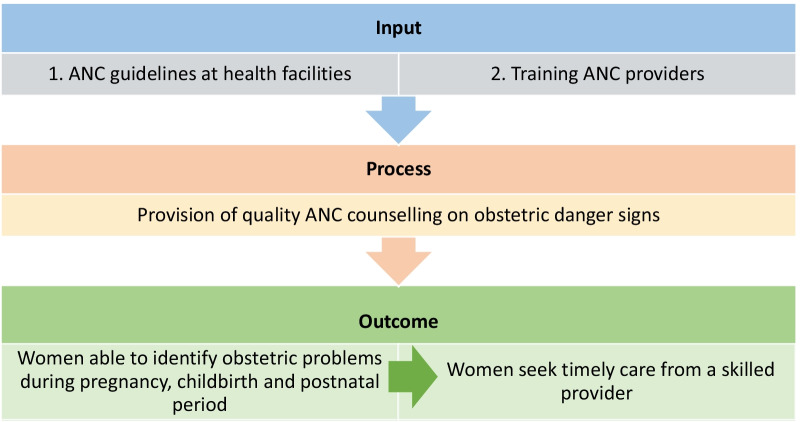
Conceptual framework of the relationship of national ANC guidelines and ANC provider uptake of training with quality ANC counselling and outcomes

To illustrate the relationship between ANC guidelines and ANC provider training, quality counselling and its outcome, we constructed a conceptual framework (see Fig. 2) based on a review of available literature [46, 47]. The improvement of ANC counselling in quantity and quality positively influences a woman’s ability to recognise obstetric danger signs early and seek timely care [16, 29]. We defined quality ANC counselling in this study as the conversation an ANC provider has with a woman concerning problems that could arise during pregnancy, childbirth and the postpartum period, as recommended by WHO [20], and what the woman should do if she experiences these. This includes counselling each woman on obstetric danger signs, the importance of nutrition during pregnancy and following childbirth, childcare and breastfeeding, and family planning. While the quantity of ANC can be defined in terms of timing and the number of visits, it can vary depending on contextual differences [48]. For example, the 2016 WHO ANC guideline recommends a minimum of eight antenatal care contacts [20], whereas the recommended minimum number of ANC visits in Ethiopia is four [13]. It is beyond the scope of the present study to focus on the quantity of ANC. The present study only focuses on the relationship between ANC counselling and the availability ANC guidelines and provider training. The relationship between ANC counselling, maternal health literacy and women’s decision to give birth at a health facility is addressed in another study (the results not provided). Guidelines can be defined as “systematically developed statements to assist practitioner and patient decisions about appropriate healthcare for specific clinical circumstances” [49]. ANC guidelines are protocols that included details on managing common problems during pregnancy.

Counselling on obstetric danger signs for pregnant women during ANC was an outcome variable measured using data from an observation checklist that recorded if the ANC provider counselled each pregnant woman on each obstetric danger. The 2014 ESPA + survey observation checklist included seven obstetric danger signs (vaginal bleeding, fever, blurred vision and severe headache, swollen hands and face, reduced or absence of foetal movement, difficulty breathing, and convulsion/ loss of consciousness). In addition, the checklist used the statement “*Any other symptoms or problems the client thinks might be related to this pregnancy*” to address if each pregnant woman received counselling on any other pregnancy-related problems. A score of “1” was assigned to each obstetric danger sign if a woman was counselled; otherwise “, 0”. Thus, the counselling score ranges from 0 to 8. The score represents the number of obstetric danger signs counselled for a woman [44]. Facility-level availability of ANC guideline (defined as 1 if it was available, otherwise 0) and ANC providers’ uptake of in-service/a refreshment training on ANC in the last 24 months preceding the 2014 ESPA + survey (defined as 1 if ANC provider took training, otherwise 0) are treatment variables. The covariates included women’s age, educational level and obstetric history (gestational age, number of ANC visits a woman had, and the number of lifetime pregnancy); health facility characteristics (health facility type, managing authority, and location); and healthcare provider characteristics (gender and profession) (Table 1).

**Table 1:** Covariates included in PSM to estimate the effect of ANC guideline and ANC providers’ training on ANC counselling

Variables	Categories
Client related variables	
Age in years NB: *Age was in the continuous form to estimate the effect of provider training on counselling*	[1] 15–20 [2] 21–25 [3] 26–30 [4] ≥ 31
School attendance	[1] Yes [0] No
Obstetric history	
Trimester NB: *It was in the continuous form to estimate the effect of provider training on counselling*	[1] First trimester [2] Second trimester [3] Third trimester
ANC visit number	[1] First visit [0] Re-visit
Number of pregnancies	[1] First pregnancy [0] Not first pregnancy
Facility related variables	
Facility location	[1] Urban [0] Rural
Facility type	[1] Hospital or Health Centre [0] Clinic
Managing authority of the facility	[1] Government [0] Other than government
Zonal level supervision	[1] Yes [0] No
Health information system available	[1] Yes [0] No
ANC provider related	
Type of profession	[1] Nurse or Midwife [0] Others

### Statistical analysis

Given the non-experimental nature of the 2014 ESPA + survey data, the analysis involved a propensity score matching (PSM) method [50]. Introduced in the early 1980s, the propensity score matching method is an approach to analyse the effect of an intervention in observational studies. Its use in observational studies mimics a randomised controlled trial, creating a sample of units or study population that received the treatment and comparable on all observed characteristics with a sample of units that did not receive the treatment. Its purpose is to balance the distribution of observed baseline characteristics between the treated or exposed and untreated or unexposed group [51, 52], and therefore to reduce bias due to confounding. This method allowed us to construct treatment and control group of pregnant women who are matched by their observed baseline characteristics for each treatment variable (i.e., (1) national ANC guidelines and (2) ANC providers’ in-service training). Then we estimated the effect of each treatment variable on the outcome variable (number of obstetric danger signs addressed in counselling) [50, 52].

### Steps involved in estimating propensity scores and the treatment effect

We calculated propensity scores for each treatment variable using Stata user-written command (psmatch2) [53]. We used the logit model for each treatment variable to identify the probability of the study participants receiving treatment conditional on the observed covariates (the propensity score) [54].

Firstly, we identified the covariates to estimate the propensity score. The covariates included in the propensity score model were grouped into four categories: women’s socio-demographic characteristics, [2] obstetric history, [3] health facility characteristics, and ANC provider characteristics (Table 1). The selection of these covariates was based on their relationship with the outcome variable (counselling on obstetric danger signs) [55]. Including the covariates that are related to the outcome variable and those that may or may not be related to the exposure variable provides a precise estimation of effect size [56]. For example, women’s age and educational level influence women’s reception of counselling but do not influence the availability of national ANC guideline at a health facility [35]. Likewise, some healthcare provider characteristics such as the type of profession may affect the ANC provider’s reception of training and the performance in counselling but do not influence the availability of ANC guidelines [33, 35]. On the other hand, the characteristics of the health facility, such as being a public or a private health facility, may be related to the availability of national ANC guidelines and staff training [40]. We identified the covariates through a systematic review of literature on ANC counselling and maternal health literacy in low and lower-middle-income countries (25 Under review)*.* We iteratively included the covariates in the propensity score model until we achieved an acceptable level of balance [51, 55] and excluded variables that showed unsatisfactory balanced property and variables which could be affected by the treatment [55, 57, 58].

Secondly, we calculated the standardised difference in proportions and means to assess whether propensity score matching has removed the differences in observed baseline characteristics between treated and control groups [59]. We chose a maximum of 10% standardised differences in the means and proportions as criteria to define covariates are balanced between the treated and control study participants [55].

Lastly, to determine the effects of treatment variables on the outcome variable, we chose the nearest neighbour one-to-one matching without replacement. The caliper distance of the propensity scores between the treated and control groups was set to be 0.01 and 0.002 for national ANC guideline and ANC provider training, respectively. While there is no consensus on the value of caliper distance [60], we decided on the width of the caliper by observing that the kernel density plots between the treated and control study participants are closely similar after matching (see figures in Additional file 1) while retaining the optimum sample [61]. The smaller the width of the caliper, the closer the match between the treated and control groups despite an increased drop in the sample [62]. The results are reported according to reporting guidelines on PSM [63].

## Results

Of the 1725 pregnant women included in the analysis, 713 (41.5%) attended ANC at health facilities with national ANC guidelines, and 815 (47.3%) received ANC service from a provider who took ANC related in-service training in the last 24 months preceding the ESPA + survey 2014.

### National ANC guideline and ANC counselling

Table 2 shows that most study participants were aged between 21 and 25 years and were in the third trimester. About two-thirds of the study participants attended school. Slightly more than one-third were pregnant for the first time, about 58% were in the third trimester, and less than half of them attended ANC for the first time.

**Table 2:** Comparison of the standardised differences of baseline characteristics before and after matching for facility-level availability of the national ANC guideline

Variable	Unmatched	Per cent (%)		Bias (%)	Reduced bias (%)	t-test	
	Matched	Treated	Control			t	p
Age category in years: 15–20	Unmatched	21.1	25.3	− 10.0	88.9	− 2.04	0.042
	Matched	23.5	24.0	− 1.1		− 0.20	0.844
21–25	Unmatched	33.0	31.0	4.3	− 55.7	0.89	0.376
	Matched	34.5	31.4	6.7		1.19	0.234
26–30	Unmatched	34.1	30.2	8.4	0.1	1.73	0.085
	Matched	28.7	32.7	− 8.3		− 1.52	0.129
≥30	Unmatched	11.7	13.5	− 5.2	26.9	− 1.05	0.293
	Matched	13.2	11.9	3.8		0.68	0.499
Ever attended school	Unmatched	71.7	66.5	11.3	78.9	2.30	0.021
	Matched	69.5	68.4	2.4		0.42	0.672
Gestational age: First trimester	Unmatched	7.3	5.7	6.2	79.5	1.28	0.200
	Matched	5.7	5.3	1.3		0.25	0.806
Second trimester	Unmatched	35.7	37.2	− 3.2	− 50.6	− 0.66	0.507
	Matched	35.3	37.7	− 4.9		− 0.87	0.383
Third trimester	Unmatched	57.1	57.0	0.1	− 6040.7	0.01	0.989
	Matched	59.0	57.0	4.1		0.74	0.461
First-time pregnancy	Unmatched	33.7	33.9	− 0.6	− 698.4	− 0.13	0.899
	Matched	36.6	34.2	5.0		0.88	0.380
ANC visit is first	Unmatched	44.3	47.1	− 5.6	55.0	− 1.15	0.252
	Matched	44.1	45.4	− 2.5		− 0.45	0.652
Facility is urban	Unmatched	83.4	70.7	30.4	97.5	6.12	0.000
	Matched	81.3	81.0	0.8		0.14	0.886
The facility owner is government	Unmatched	87.1	82.4	13.3	50.5	2.68	0.007
	Matched	85.9	88.2	− 6.6		− 1.25	0.211
Facility is hospital or health centre	Unmatched	97.1	95.6	7.6	55.8	1.53	0.127
	Matched	96.9	97.5	− 3.4		− 0.68	0.499
Facility is supervised at the zonal level	Unmatched	47.9	55.4	− 15.3	85.6	− 3.13	0.002
	Matched	53.2	52.1	2.2		0.39	0.695
ANC provider is Nurse or Midwife	Unmatched	87.1	83.8	9.6	95.3	1.94	0.052
	Matched	85.9	85.7	0.4		0.08	0.936
HMIS is available	Unmatched	97.2	93.5	17.8	95.8	3.52	0.000
	Matched	96.9	96.7	0.7		0.16	0.874

The nearest one-to-one matching without replacement yielded 1,274 samples comprised of 637 women who received ANC at health facilities with national ANC guidelines (treated) and 637 women who received ANC at health facilities with no national ANC guideline (control).

We found that the absolute value of the calculated standardised difference in means and proportions of each variable after matching is less than 10%, indicating the presence of a balanced match on the observed baseline characteristics between treated and control groups.

**Fig. 3:**
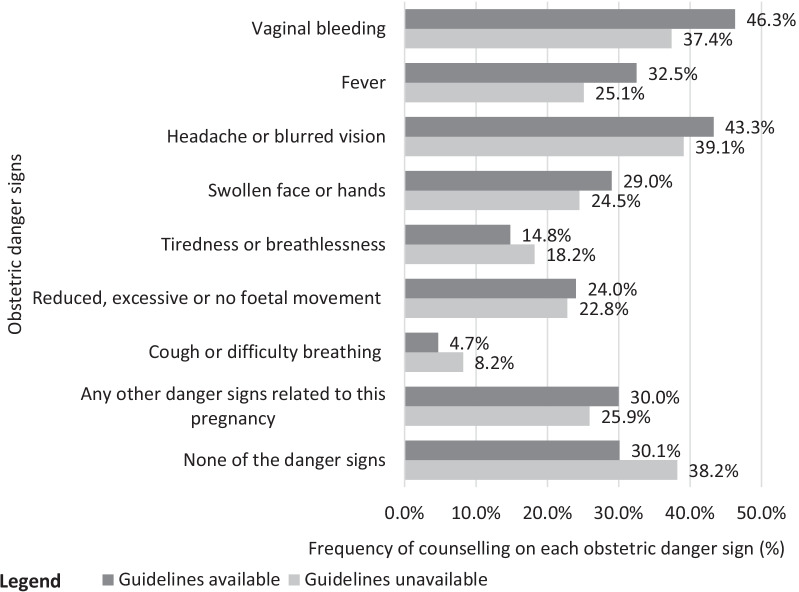
Comparison of counselling on each obstetric danger sign by facility-level availability of national ANC guidelines

Figure 3 presents the frequency of counselling on each obstetric danger sign for pregnant women who received ANC at health facilities with national ANC guidelines compared to those who received ANC at health facilities without guidelines. The number of pregnant women who received ANC counselling on each obstetric danger sign differs by the facility-level availability of the national ANC guidelines. The frequency of counselling on most obstetric danger signs was higher at health facilities with ANC guidelines than without guidelines. Vaginal bleeding was the most counselled obstetric danger sign in the treatment group (46.3%). In contrast, headache or blurred vision was the most counselled obstetric danger sign in the control group (39.1%). On the contrary, cough or difficulty breathing was the least counselled obstetric danger sign for pregnant women in the treatment (4.7%) and control (8.2%) groups. Thirty per cent of women in the treatment group and 38.2% of women in the control group did not receive counselling on any obstetric danger sign.

After matching the study participants by the observed baseline characteristics, we found that facility-level ANC guidelines increased the average number of obstetric danger signs included in counselling women by 24% (95% CI: 12%—35%) (Table 3). A Wilcoxon signed-rank test for matched pairs [64] also indicated that the observed difference in obstetric danger sign counselling related to the availability of guidelines is statistically significant (z = -2.301, p = 0.021).

**Table 3:** Average treatment effect of facility-level availability of national ANC guideline on ANC counselling on obstetric danger signs

Outcome variable	Sample	Treated	Control	Difference	S.E
The mean number of danger signs counselled	Unmatched	2.27	1.93	0.34	0.101
	ATT	2.25	2.01	0.24	0.117

### ANC providers’ uptake of training and ANC counselling

The second treatment variable in this study was the ANC provider’s reception of ANC related in-service training in the last 24 months preceding the ESPA + survey 2014. Matching yielded thirteen hundred and sixty-eight pregnant women, in which 684 women received ANC from trained providers, other 684 women received ANC from untrained providers.

Table 4 shows that the mean age of the study participants was 25 years. About 70% of the women had attended school. The average gestational age of the study participants was 27 weeks. One-third of the study participants were pregnant for the first time.

**Table 4:** Comparison of the standardised differences of baseline characteristics before and after matching for ANC providers’ uptake of refresher training

Variable	Unmatched	Per cent (mean)		Bias (%)	Reduced bias (%)	t-test	
	Matched	Treated	Control			t	p
Age in years (mean)	Unmatched	25.2	25.4	− 3.7	38.0	− 0.77	0.439
	Matched	25.3	25.2	2.3		0.43	0.665
School attendance	Unmatched	68.5	68.9	− 0.9	− 337.1	− 0.19	0.846
	Matched	68.9	70.8	− 4.1		− 0.77	0.444
Gestational age in weeks (mean)	Unmatched	27.5	27.1	4.7	20.1	0.97	0.335
	Matched	27.0	27.3	− 3.7		− 0.69	0.490
First-time pregnancy	Unmatched	37.3	30.6	14.3	100.0	2.97	0.003
	Matched	34.3	36.4	0.0		0.00	1.000
ANC visit is first	Unmatched	45.2	46.7	− 3.1	− 22.6	− 0.64	0.519
	Matched	48.0	46.1	3.8		0.70	0.482
Facility is urban	Unmatched	82.6	70.0	29.9	94.2	6.16	0.000
	Matched	80.0	79.2	1.7		0.34	0.737
Facility owner is government	Unmatched	82.9	85.6	− 7.3	34.0	− 1.51	0.129
	Matched	86.0	84.2	4.8		0.91	0.363
Facility is hospital or health centre	Unmatched	95.2	97.1	− 10.1	24.2	− 2.10	0.036
	Matched	98.4	97.0	7.6		1.79	0.074
Facility is supervised at the zonal level	Unmatched	50.7	53.7	− 6.1	9.3	− 1.27	0.204
	Matched	53.1	50.3	5.6		1.03	0.304
ANC provider is Nurse or Midwife	Unmatched	83.8	86.3	− 7.2	100.0	− 1.50	0.134
	Matched	84.8	84.8	0.0		0.00	1.000

**Fig. 4:**
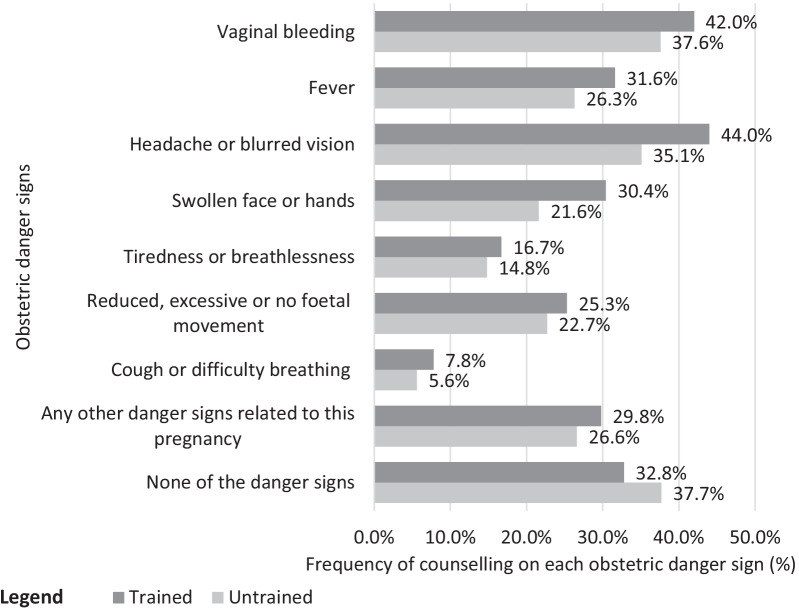
Comparison of counselling on each obstetric danger sign with by ANC providers’ receipt of refresher training

Figure 4 shows that headache or blurred vision was the most counselled obstetric danger sign for pregnant women in the treatment group (44.0%). In contrast, the most counselled obstetric danger sign for pregnant women in the control group was vaginal bleeding (37.6%). The least counselled obstetric danger sign was cough or difficulty breathing in both the treatment (7.8%) and control (5.6%) groups. Thirty-two point eight per cent of women in the treatment group and 37.7% of women in the control group did not receive counselling on any obstetric danger sign.

Table 5 shows that ANC providers’ uptake of ANC related in-service training in the last 24 months increased the mean number of obstetric danger signs included in counselling pregnant women by 37% (95% CI: 26%—48%). This increment was statistically significant in the Wilcoxon signed-rank test for paired sample (z = -3.212, p = 0.001).

**Table 5:** Average treatment effect of the ANC providers’ uptake of ANC related refresher training on ANC counselling on obstetric danger signs

Outcome variable	Sample	Treated	Control	Difference	S.E
The mean number of danger signs counselled	Unmatched	2.25	1.90	0.35	0.099
	ATT	2.27	1.90	0.37	0.112

## Discussion

This PSM study using the ESPA + 2014 data showed that the availability of national ANC guidelines at the facility level significantly increased the average number of obstetric danger signs discussed with pregnant women during ANC counselling by 24%. Similarly, providing ANC related in-service training for the ANC providers significantly increased the average number of obstetric danger signs discussed with women received during ANC counselling by 37%. While some women received no counselling on any danger signs, others received no counselling on a particular danger sign. Cough or difficulty breathing were found to be the least discussed danger signs. These danger signs are very relevant in the context of the current COVID-19 pandemic. A cough or difficulty breathing can signify both pregnancy-related complications and COVID-19 infection. Our study shows that a higher percentage of counselling sessions on most obstetric danger signs were at health facilities with guidelines and trained providers. The findings demonstrate the importance of the national ANC guidelines and in-service training for the ANC providers in counselling each pregnant woman on every obstetric danger sign.

The results are consistent with study findings from other countries that have shown an increase in counselling on obstetric danger signs due to guidelines or job aids and provider training [41–43]. The use of ANC counselling job aids in Benin resulted in a 26% increase in counselling on obstetric danger signs [42]. Similarly, in Tanzania, more women in the intervention group involving ANC counselling job aids reported counselling reception on obstetric danger signs than women in control (no job aid intervention) [41]. In Guatemala, there was a 15% increase in women who received counselling on obstetric danger signs following the use of counselling guidelines [43].

ANC guidelines increase counselling on obstetric danger signs by enhancing provider-recipient communication for effective counselling on obstetric danger signs. This is attributed to the printed information on the ANC guidelines outlining clear instructions that support ANC providers approaching pregnant women and discussing pregnancy-related topics [24, 65]. Guidelines also contain a list of pregnancy-related complications and danger signs to be discussed with a woman. Providers can discuss each of the danger signs with each pregnant woman based on the instructions in the guideline and let them know what to do if the danger signs occur [35, 38]. ANC guidelines are also essential to reduce the time that the providers would typically spend thinking about the types of prenatal healthcare that should be provided to the expectant mother. As a result, this maximises the probability of counselling on each obstetric danger sign [41–43, 66].

In the GANC model, ANC guidelines consist of a list of instructions on a range of pregnancy-related topics, including danger signs, that can be used to facilitate communication between the provider and recipients [29–31]. The implementation of GANC involves developing country-context guidelines and training facilitators (the ANC providers) on how to use these guidelines to facilitate women’s discussion in a group setting. The provider uses these guidelines to promote discussion that encourages women to share their experiences and learn from each other [29–31, 67, 68]. While this model may be useful to improve the ANC counselling on danger signs, implementing GANC might be challenging in the context of the coronavirus pandemic due to the increased risk of COVID-19 infection in a group setting [69]. Thus, the traditional one-to-one ANC would be preferred to apply physical distancing in reducing COVID-19 transmission [70]. One-to-one ANC counselling could be as effective as GANC in achieving successful counselling on obstetric danger signs if guidelines are in place for use by trained providers [42, 43].

Another facility-level factor influencing ANC counselling that we examined in this study is ANC provider uptake of ANC related in-service training. Consistent with the findings of other studies [42, 71, 72], our study findings showed that women have a 37% increased chance of receiving counselling on obstetric danger signs if the providers had ANC related in-service training in the last 24 months.

Appropriate and acceptable care tailored to the pregnant woman’s socio-cultural context requires providers to be clinically competent, motivated and available [73, 74]. Training, mentoring, and supportive supervision are among the strategies that make the ANC provider capable and motivated [66, 75]. Provider training includes pre-service training and in-service training. In-service training is a low-cost option to refresh provider knowledge and skills [76, 77], ensuring their competencies are up-to-date [38, 66]. Moreover, in-service ANC-related training is vital to address the emerging healthcare needs of pregnant women and to take their socio-cultural context into account [78]. With up-to-date knowledge and skills on ANC, providers can communicate effectively with pregnant women and understand their needs, beliefs, and values. These skills and understanding of the socio-cultural context of women enable providers to assess and identify problems and assist women in making informed decisions. Ethiopia is the home of people with multiple socio-cultural characteristics that play an important role in healthcare provision [79]. Provider in-service training helps the provider understand and respect these socio-cultural attributes of women to establish interactive communication [80]. Interactive provider–client communication that takes social and cultural norms into account gives pregnant women an extra opportunity to discuss a range of pregnancy-related topics, including how to recognise danger signs and how and where to seek treatment [24, 81]. In-service training also increases providers’ motivation (Momanyi et al. 2016) and confidence [82]. A motivated and confident ANC provider makes informed clinical decisions [83] and takes professional responsibility and accountability to provide the highest possible quality care [84].

Other interventions that could improve the quality of ANC counselling include systematic quality improvements at the facility level (e.g., audits and feedback), support to enhance quality infrastructure, and community participation [75, 81, 85]. A study in Malawi found that providers felt happier and motivated, and patient satisfaction increased after implementing monthly supportive staff meetings. The monthly meetings involved sharing stories that involved identifying best practices and developing plans to implement these [86]. These facility-level quality improvement strategies could also be supported by community-based interventions [65], such as implementing social and behavioural change communication. Involving community leaders, such as religious leaders, in disseminating health messages regarding pregnancy, childbirth, postnatal and newborn care, has been shown to increase women’s knowledge of obstetric danger signs and maternal healthcare service reception [43, 85].

## Strength and limitations

Applying the PSM method in an observational data (the 2014 ESPA + survey data) enabled us to estimate the unconfounded treatment effects of ANC guidelines and provider in-service training on the provision of counselling on obstetric danger signs. However, this study has some limitations. The PSM method only adjusts for measured covariates. Therefore, this study does not guarantee the elimination of bias due to unmeasured covariates. The PSM also excluded unmatched samples that may be systematically different from the matched samples, which could affect the representativeness of the study population. We acknowledge the findings in this study may or may not directly represent the current ANC counselling practice in Ethiopia because the 2014 ESPA + survey was undertaken seven years ago as of 2021. Thus, care should be taken to interpret the results. However, as the 2014 ESPA + survey is a national representative data, the findings of this study are comprehensive to provide policymakers with quality insights to improve ANC quality. Additionally, the providers’ uptake of in-service training was self-reported, which might have been affected by the recall and social desirability biases. Similarly, this study did not examine whether ANC providers consistently used the ANC guidelines during ANC counselling.

## Conclusion and recommendations

We found that counselling pregnant women about obstetric danger signs during their ANC contacts is not universally practised. Nearly one in three women do not receive counselling on any obstetric danger sign. The availability of facility-level national ANC guidelines and in-service training for the ANC providers are positively and significantly associated with the number of obstetric danger signs discussed with women during ANC counselling. Decision-makers need to prioritise funding and policy to build supportive environments to ensure each health facility has guidelines and continuous in-service training programs are available for every ANC provider. Further research is required to understand whether ANC guidelines are consistently used during ANC service provision and examine whether this is associated with the quality of ANC counselling.

## Supplementary Information

**Additional file 1.** Figures illustrating the presence of match onobserved covariates between treated and control groupsbefore and aftermatching for both treatment variables (ANC guidelines andANC providers’training).

## Data Availability

All data related to the study findings are incorporated into the article and its online Additional file 1. Access to the 2014 ESPA + survey data requires permission from the EPHI.

## Abbreviations

ANC: Antenatal care
ATT: Average treatment in treated
COVID-19: Coronavirus disease
EPHI: Ethiopian Public Health Institute
ESPA +: Ethiopian Service Provision Assessment Plus
GANC: Group antenatal care
HSTP: Health Sector Transformation Plan
HMIS: Health management information system
MMR: Maternal mortality ratio
PSM: Propensity score matching
SDG: Sustainable development goals
SSA: Sub-Saharan Africa
UTS: University of Technology Sydney
WHO: World Health Organization
