# Correction: The effect of national antenatal care guidelines and provider training on obstetric danger sign counselling: a propensity score matching analysis of the 2014 Ethiopia service provision assessment plus survey

**DOI:** 10.1186/s12978-022-01483-x

**Published:** 2022-08-17

**Authors:** Tebikew Yeneabat, Andrew Hayen, Theodros Getachew, Angela Dawson

**Affiliations:** 1 Department of Midwifery, College of Health Sciences, Debre Markos University, Debre Markos, Ethiopia; 2 School of Public Health, Faculty of Health, University of Technology Sydney, Sydney, Australia; 3 Health System and Reproductive Health Research Directorate, Ethiopian Public Health Institute, Addis Ababa, Ethiopia

## Correction to: Reproductive Health (2022) 19:132 https://doi.org/10.1186/s12978-022-01442-6

After publication of this article [1], the authors reported that in the section ‘Sample size and selection process’ in the first sentence, the number ‘1237’ should have read ‘1327’.

Moreover, reference [50] should have been replaced by [53]. References [54–87] were renumbered.

The original article [1] has been corrected.
