# Supplementary material for: The effect of national antenatal care guidelines and provider training on obstetric danger sign counselling: a propensity score matching analysis of the 2014 Ethiopia service provision assessment plus survey
Source: Reprod Health. 2022 Jun 6;19:132. doi: 10.1186/s12978-022-01442-6 (PMC9167913; doi:10.1186/s12978-022-01442-6)
Supplement: Supplementary file 1 — Additional file 1. Figures illustrating the presence of match onobserved covariates between treated and control groupsbefore and aftermatching for both treatment variables (ANC guidelines andANC providers’training). [file 12978_2022_1442_MOESM1_ESM.docx]

**Additional file 1 Figures illustrating the presence of match on observed covariates between treated and control groups before and after matching for both treatment variables (ANC guidelines and ANC providers’ training)**


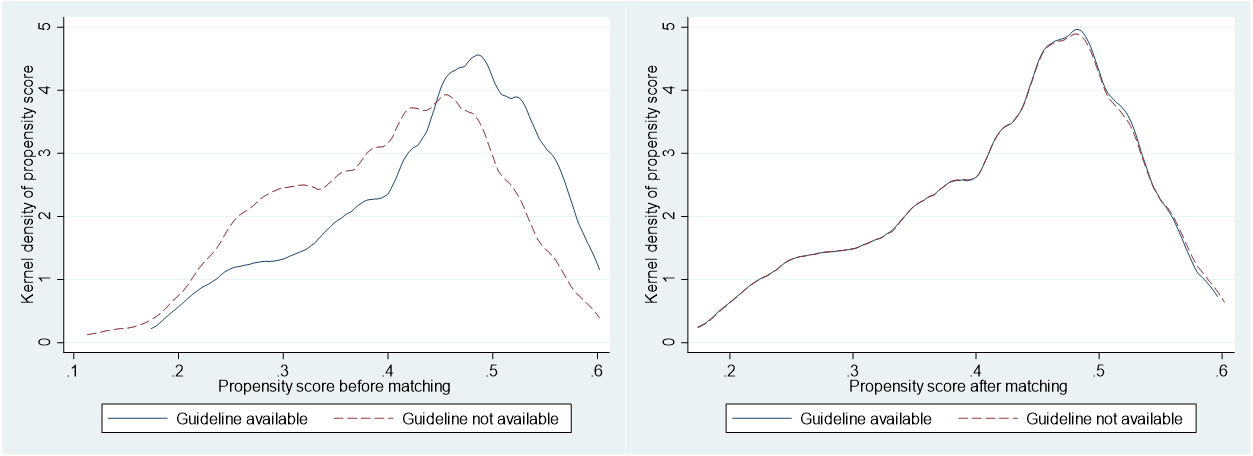


Figure I Kernel density of propensity score before and after matching for treatment ANC guideline


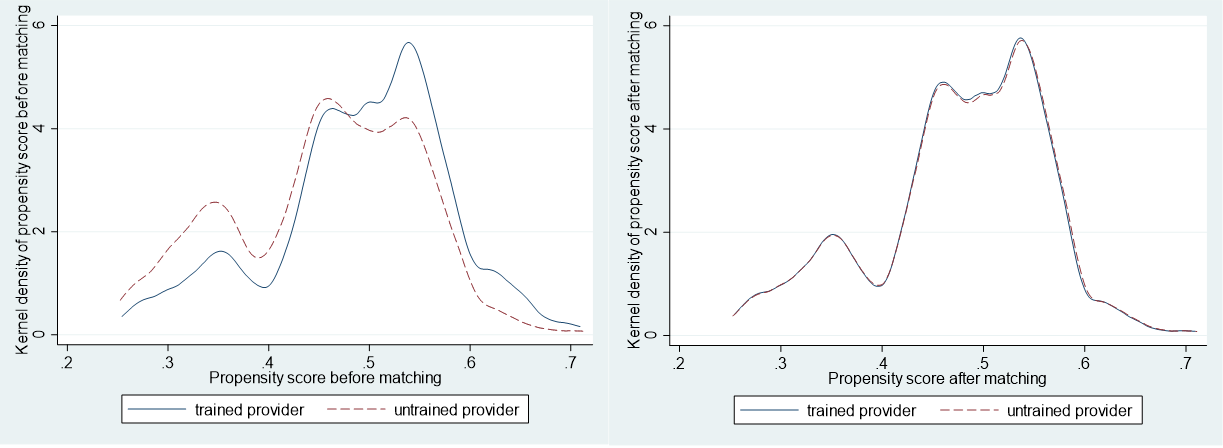


Figure II Kernel density of propensity score before and after matching for treatment providers’ training
